# Environmental sustainability of ultrasound-guided core-needle breast biopsy: a survey on current practices by the European Society of Breast Imaging (EUSOBI)

**DOI:** 10.1186/s13244-026-02215-6

**Published:** 2026-02-03

**Authors:** Andrea Cozzi, Serena Carriero, Maria Adele Marino, Simone Schiaffino, Fleur Kilburn-Toppin, Matthew G. Wallis, Paola Clauser, Michael H. Fuchsjäger, Elisabetta Giannotti

**Affiliations:** 1 Imaging Institute of Southern Switzerland (IIMSI), Ente Ospedaliero Cantonale (EOC), Lugano, Switzerland; 2 Radiology and Interventional Radiology, Fondazione IRCCS Ca’ Granda Ospedale Maggiore Policlinico, Milano, Italy; 3 Department of Biomedical Sciences and Morphologic and Functional Imaging, Università degli Studi di Messina, Messina, Italy; 4 Faculty of Biomedical Sciences, Università della Svizzera Italiana, Lugano, Switzerland; 5 Cambridge Breast Unit, Cambridge University Hospital NHS Foundation Trust, Cambridge, United Kingdom; 6 Division of General and Pediatric Radiology, Department of Biomedical Imaging and Image-Guided Therapy, Medical University of Vienna, Vienna, Austria; 7 Division of General Radiology, Department of Radiology, Medical University Graz, Graz, Austria

**Keywords:** Breast neoplasms, Image-guided biopsy, Sustainable development, Environment and Public Health

## Abstract

**Objectives:**

In the context of a global appraisal of the environmental impact of radiology, this survey among members of the European Society of Breast Imaging (EUSOBI) investigated procedural aspects of ultrasound-guided core-needle breast biopsy that may impact its environmental sustainability.

**Materials and methods:**

A 25-item online questionnaire, developed by a panel of nine breast imaging experts, was distributed from September 25th to December 25th, 2024, within the EUSOBI mailing list and social media platforms. The survey investigated materials routinely used for ultrasound-guided core-needle biopsies, waste disposal practices, the relationship between perceived procedural hygiene levels and self-reported frequency of post-procedural infectious complications, and results’ communication methods. Replies were analysed with descriptive and non-parametric statistics.

**Results:**

Among the 787/823 respondents (95.6%) who routinely perform ultrasound-guided core-needle biopsy, most (460/787, 58.4%) perceived to attain aseptic conditions, without significant associations (*p* = 0.334) of hygiene levels with post-procedural infectious complications (never seen by 549/776 respondents, 70.7%). For most disposable materials, the majority of respondents used no more than one unit per procedure, including sterile gloves (551/787, 70.0%), sterile drapes (651/787, 82.7%), and sterile gel packets (729/787, 92.6%), also avoiding to use prepackaged biopsy kits (424/787, 53.9%). However, most respondents did not use recycling bins (404/787, 51.3%) and employed at least one resource-intensive modality to communicate benign results (in-person or by letter, 584/787, 74.2%).

**Conclusion:**

Procedural aspects of ultrasound-guided core-needle biopsy carrying an environmental impact vary widely. In the absence of significant associations between perceived hygiene levels and post-procedural infectious complications, resource-intensive habits could be safely streamlined to improve sustainability.

**Critical relevance statement:**

This EUSOBI survey demonstrates that, despite widely varying procedural aspects in ultrasound-guided core-needle breast biopsy, higher perceived sterility levels are not associated with fewer infections, highlighting opportunities to safely reduce resource use and environmental impact.

**Key Points:**

- This EUSOBI survey investigated how procedural habits and the use and amount of material in ultrasound-guided core-needle breast biopsy impact its environmental sustainability.
- Procedural aspects varied widely among the 787/823 respondents who routinely perform ultrasound-guided core-needle breast biopsy.
- While some economically driven sustainable behaviours are already in place, there are several opportunities to reduce materials use and waste.
- As no association was found between perceived hygiene levels and post-procedural infections, resource-intensive hygiene-related practices could be streamlined to improve sustainability.

**Graphical Abstract:**

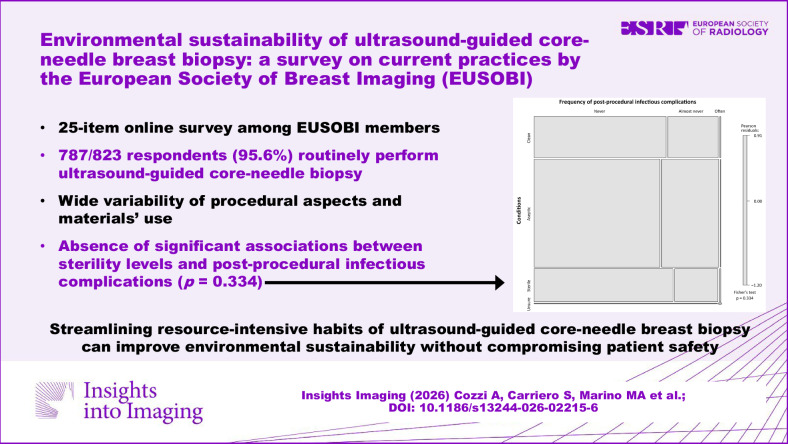

## Introduction

While the industry and energy sectors have traditionally been the focus of research and practical interventions for environmental sustainability [1, 2], the healthcare sector—responsible for an estimated 5% of global greenhouse gas emissions—is now recognised both as a contributor to environmental degradation and as a potential leader in driving change [3–5]. Across different medical specialities, including radiology, there is a growing imperative to rethink daily clinical activities through the lens of environmental responsibility [4–10].

Within this context, breast imaging has a particularly relevant position, as it combines high patient volumes, standardised workflows, and frequent use of disposable materials. Ultrasound-guided core-needle biopsy is one of the most performed procedures in breast care, having largely replaced surgical biopsy by offering a safe, minimally invasive, and cost-effective option [11, 12].

There is substantial variability in how ultrasound-guided core-needle biopsy is performed across different centres [11, 12]. Despite it being a low-risk procedure with minimal bleeding [13–15] and an extremely low infection rate—reported to be less than 0.3% [16], pre-procedural and peri-procedural hygiene practices vary widely, with some operators adopting fully sterile techniques and others opting for aseptic or clean procedures. There is often limited awareness of the rationale that underpins the choice between these different hygienic approaches, also considering that several socioeconomic factors—including the availability and cost of disposable materials—might influence this choice at national, regional, and even institutional levels [11, 13, 16, 17].

Although no robust evidence has ever been presented to support the necessity of rigorous sterile precautions for core-needle breast biopsy [11, 13], the procedure commonly involves an abundant and non-standardised use of single-use disposable materials, such as gloves, sterile drapes, syringes, needles, ultrasound probe covers, gauze swabs, plasters, and bandages. All these materials, especially in interventional procedures, represent a major source of biomedical waste [18–20], with far-reaching environmental implications [21, 22].

This survey, promoted by the European Society of Breast Imaging (EUSOBI), aims to provide a comprehensive overview—both from a qualitative and quantitative point of view—of several aspects of ultrasound-guided core-needle breast biopsy that carry a potential environmental impact, in order to identify opportunities to minimise waste and prepare for future recommendations on sustainable breast imaging.

## Materials and methods

As this online survey was fully anonymous and implied voluntary consent from the participants, the Ethics Committee of the institution of the senior author (Cambridge University Hospital NHS Foundation Trust, Cambridge, United Kingdom) required no specific approval. The questionnaire (reported in the Supplementary Material) was developed by a panel of nine experts in breast cancer imaging (S.C., A.C., S.S., M.A.M., F.K.-T., M.G.W., P.C., M.H.F., and E.G.) representing different experience levels and scopes of activity in breast imaging (e.g., public, private and university hospitals) across Europe. The panellists discussed and agreed upon questions about materials and practices that were deemed to be common for ultrasound-guided core-needle breast biopsy across different practice settings and countries, while also aiming to build a questionnaire that could be completed in about 10 minutes to encourage participation and minimise incomplete responses. Ultimately, the questionnaire comprised 25 questions divided into two main sections: the first focusing on demographics and clinical practice settings of the respondents, and the second on their specific experience with ultrasound-guided core-needle breast biopsy, including procedural habits and resource utilisation. Two different pathways were designed, one for respondents who declared they performed core-needle breast biopsy and one for those who claimed they did not perform any. For those responders who indicated they did not perform biopsies, the questionnaire ended, while the full questionnaire was accessible only to those who performed core-needle breast biopsies in their daily practice. The survey was conducted anonymously.

First, the respondents who declared to perform ultrasound-guided core-needle breast biopsies were asked to express their opinion on how they would define their way of performing core-needle biopsy according to three different scenarios: (i) *sterile*, i.e., performed in a completely sterile environment; (ii) *aseptic*, i.e., using aseptic techniques (including sterile gloves) to minimise contamination; (iii) *clean*, i.e., maintaining basic cleanliness without attaining the levels required by asepsis or sterility.

Then, the survey explored the type and amount of material used during core-needle breast biopsy, accounting for their total number (per operator and assistant, if any, whose presence was the focus of a specific question) across all procedural steps, from operator preparation to sample collection and post-procedural care. Specific materials assessed were: sterile and non-sterile gloves, sterile keyboard covers for ultrasound systems, probe covers, sterile drapes, sterile gowns, hair caps, sterile gel, sterile and non-sterile gauze swabs, local anaesthetic vials, scalpels, plastic trays (used for disinfectant, gauzes, or sharp materials) and biopsy kits.

In addition to procedural details, the survey examined waste disposal practices, the self-reported and self-estimated perceived frequency of post-procedural infectious complications, and the methods used to communicate benign and malignant results to the patients.

The survey was disseminated through the EUSOBI official mailing list and made available on Google Forms (Google LLC) from September 25th 2024, to December 25th 2024. Two reminders were sent to all contacts in the mailing list on November 25th 2024, and on December 15th 2024. In addition to email dissemination, the survey was promoted on the official EUSOBI Instagram page through stories containing a direct link to the questionnaire. This social media promotion occurred on five separate occasions between September 25th and November 25th, 2024.

After the closure of the survey, data were exported in spreadsheets, coded wherever appropriate for multiple-choice or open-ended questions, then analysed and reported through descriptive statistics, presenting categorical data as counts and percentages. The Fisher’s exact test was used to evaluate potential associations between procedural hygiene practices and the occurrence of post-procedural infectious complications. Due to the very small number of missing replies (< 1.5% of respondents in each question), no correction for missing data was applied. All analyses were conducted with R (version 4.5.0, The R Foundation for Statistical Computing).

## Results

### Responses to the survey

The survey was completed by 823 respondents, most of whom (787/823, 95.6%) perform US-guided core-needle breast biopsy in their clinical practice. As the complete questionnaire was only available for respondents who declared to effectively perform US-guided core-needle breast biopsy, all analyses focused on these 787 respondents. Most of the respondents came from Europe (491/787, 62.4%), followed by respondents from Asia (131/787, 16.6%), the Americas (67/787, 8.5%), Africa (14/787, 1.8%), and Oceania (11/787, 1.4%), while geographic origin was not declared by 73/787 respondents (9.3%).

**Fig. 1:**
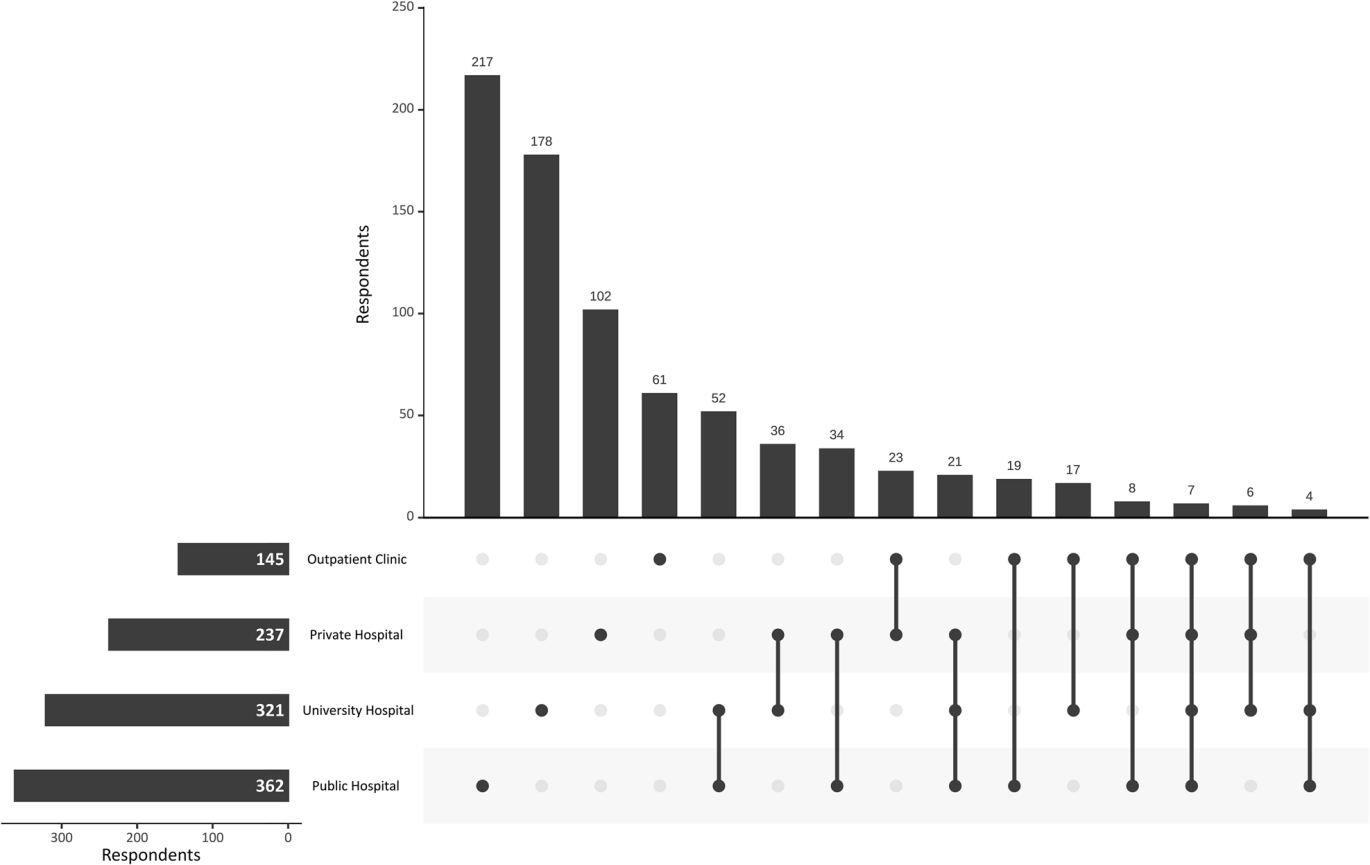
Upset plot of practice settings among respondents

Most respondents (560/787, 71.2%) declared to perform US-guided core-needle biopsy only in one of the five practice settings indicated in the survey, with public hospitals being the most common single setting (217/787, 27.6%), followed by university hospitals (178/787, 22.6%). Overall, considering multiple replies, 362/787 respondents (46.0%) declared to perform core-needle biopsies in public hospitals, 321/787 in university hospitals (40.8%), 237/787 in private hospitals (30.1%), and 145/787 (18.4%) in private outpatient settings (Fig. 1).

Most respondents declared that they aimed to perform core-needle biopsies in aseptic conditions (460/787, 58.4%), 147/787 in sterile conditions (18.7%), and 178/787 (22.6%) while maintaining only basic cleanliness without attaining sterility or asepsis; only 2/787 of respondents (0.3%) were effectively unsure about how the different choices of materials and procedural steps qualified their usual procedural pathway in one of the aforementioned categories.

More than 90% of respondents declared to perform core-needle biopsy in the presence of an assistant, who was always present in 572/787 cases (72.7%) and often present in 152/787 cases (19.3%). Only 32/787 respondents perform core-needle biopsies alone (4.1%), while the other 31/787 (3.9%) replied that an assistant was “almost never” present. If present, the assistant was involved in saving the images in 548/787 cases (69.6%), being the sole responsible for this task in 442/787 cases (56.1%). The radiologist performing the core-needle biopsy was instead involved in saving images, either with foot-switches (89/787 respondents, 11.3%) or directly touching the keyboard of the ultrasound system in 213/787 cases (27.1%). Of note, only 43 of the latter 213 respondents (20.2%) reported using at least one sterile keyboard cover.

### Materials used in ultrasound-guided core-needle breast biopsies

As shown in Tables 1–3, across all surveyed disposable materials, most respondents used no more than one item or packet for the whole procedure, also considering the presence of an assistant. In particular, only 236/787 (30.0%) respondents used more than one pair of sterile gloves, while 500/787 respondents (63.5%) used less than two pairs of non-sterile gloves. Two pairs of non-sterile gloves were used by 247/787 respondents (31.4%), among whom only 14/247 (5.7%) declared to perform the procedure alone. As already mentioned, the use of sterile keyboard covers was reported by a minority of respondents (165/787, 21.0%), with only 21/787 (2.7%) respondents using more than one (Table 1). Among the 665/787 respondents (84.5%) using a probe cover, only 58/787 (7.4%) use more than one of these items. Of note, most of these 665 respondents (447/665, 67.2%) cover the probe with sterile items (specific sterile probe covers in 381 cases and a sterile glove in 66 cases), while the remaining 218/665 respondents (32.8%) use non-sterile disposables (specific non-sterile probe covers in 190 cases and a non-sterile glove in 28 cases). At least one sterile drape is used by 65.6% of the respondents (516/787), but only 134/787 respondents (17.0%) indeed use more than one (Table 2). Conversely, the majority of respondents do not routinely use sterile gowns (562/787, 71.4%) or hair caps (614/787, 78.0%). The use of sterile gel was declared by 438/787 respondents (55.7%), but only 41/438 (9.4%) use more than one packet per procedure, with a further 16/438 respondents (3.7%) declaring to conserve opened sterile gel packets for use in multiple procedures (Table 1). Sterile gauze emerged as the most widely used type of gauze (733/787 respondents, 93.1%), with 532/787 respondents (67.6%) consuming between one and three swabs per procedure. Conversely, while 263/787 respondents use at least one swab of non-sterile gauze (33.4%), only 36/263 (13.7%) consume more than three swabs (Table 3). Finally, while the use of scalpels was frequently reported (607/787 respondents, 77.1%; Table 1), only 16 of these 607 respondents used more than one scalpel per procedure (2.6%).

**Table 1:** Amount of disposable material used for a single ultrasound-guided core-needle breast biopsy

	0	1	2	3	> 3	One for more than one procedure	No reply
Pairs of sterile gloves	137 (17.4%)	414 (52.6%)	197 (25.0%)	31 (3.9%)	8 (1.0%)	—	0 (0.0%)
Pairs of non-sterile gloves	109 (13.9%)	391 (49.7%)	247 (31.4%)	25 (3.2%)	13 (1.6%)	—	2 (0.2%)
Keyboard covers	621 (78.9%)	144 (18.3%)	14 (1.8%)	3 (0.4%)	4 (0.5%)	—	1 (0.1%)
Probe covers	122 (15.5%)	607 (77.1%)	33 (4.2%)	15 (1.9%)	10 (1.3%)	—	0 (0.0%)
Sterile gowns	562 (71.4%)	136 (17.3%)	65 (8.3%)	11 (1.4%)	9 (1.1%)	—	4 (0.5%)
Hair caps	614 (78.0%)	62 (7.9%)	72 (9.1%)	39 (5.0%)	—	—	0 (0.0%)
Sterile gel packets	348 (44.2%)	381 (48.4%)	35 (4.5%)	3 (0.4%)	3 (0.4%)	16 (2.0%)	1 (0.1%)
Plastic trays	150 (19.1%)	388 (49.3%)	129 (16.4%)	40 (5.1%)	20 (2.5%)	56 (7.1%)	4 (0.5%)
Scalpels	178 (22.6%)	591 (75.1%)	9 (1.1%)	7 (0.9%)^§^	—	—	2 (0.3%)

^§^ More than two scalpels per procedure

**Table 2:** Number of sterile drapes used for a single ultrasound-guided core-needle breast biopsy

Sterile drapes	Respondents
0	269 (34.2%)
1	382 (48.5%)
2	93 (11.8%)
3	26 (3.3%)
4	9 (1.1%)
5	4 (0.5%)
> 5	2 (0.3%)
No reply	2 (0.3%)

**Table 3:** Number of sterile and non-sterile gauze swabs used for a single ultrasound-guided core-needle breast biopsy

	Sterile gauze	Non-sterile gauze
0 swabs	51 respondents (6.5%)	515 respondents (65.4%)
1–3 swabs	532 respondents (67.6%)	227 respondents (28.8%)
3–5 swabs	129 respondents (16.4%)	20 respondents (2.5%)
5–8 swabs	54 respondents (6.9%)	11 respondents (1.4%)
8–10 swabs	11 respondents (1.4%)	4 respondents (0.5%)
> 10 swabs	7 respondents (0.9%)	1 respondent (0.1%)
No reply	3 respondents (0.4%)	9 respondents (1.1%)

**Fig. 2:**
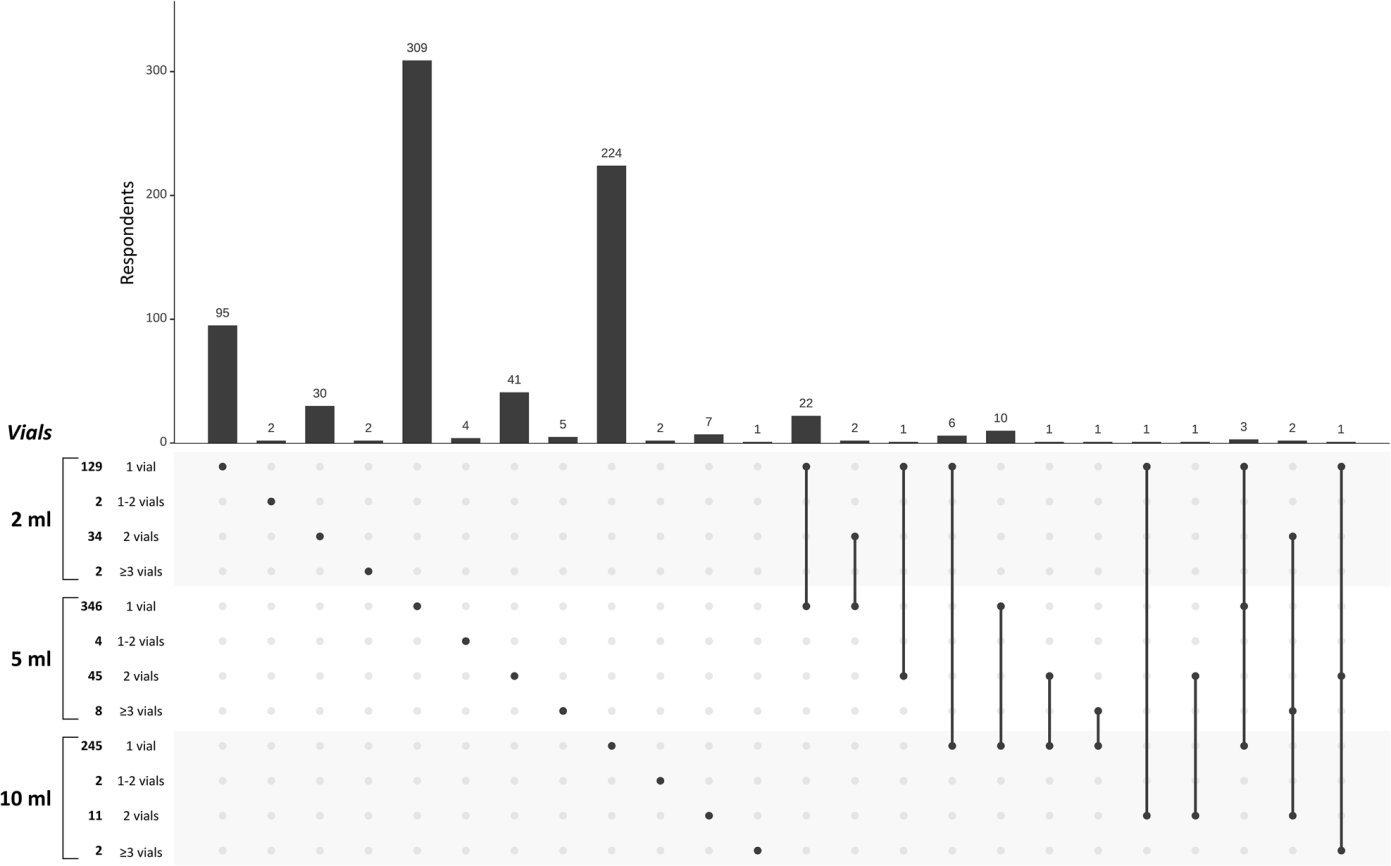
Upset plot of the respondents’ preferences for the use of anaesthetic vials for ultrasound-guided core-needle breast biopsy

As shown in Fig. 2, 5 mL anaesthetic vials resulted in being the most commonly used (403/787 respondents, 51.2%), followed by 10 mL vials (260/787 respondents, 33.0%) and 2 mL vials (167/787 respondents, 21.2%). The use of more than one vial of anaesthetic was reported by 145/787 respondents (18.4%), with only 42/787 respondents (5.4%) effectively employing—i.e., opening vials, regardless of the effectively injected quantity—more than 10 mL of anaesthetic for a single procedure.

During and after the procedure, the use of plastic trays (i.e., containers for disinfectants, garments, cutters, etc., excluding the specimen containers) remains common (633/787 respondents, 80.4%), but confined to one per procedure in 388/633 cases (61.3%) and one for more than one procedure in 56 further cases (8.8%; Table 1). However, the number of respondents declaring the use of more than one plastic tray (189/633 cases, 29.9%) remains higher than the number of respondents who do not use them (150/787, 19.1%). Finally, most respondents declared not using a recycling bin for the disposal of non-sharp tools (404/787, 51.3%) and not to routinely use prepackaged biopsy kits (424/787, 53.9%).

### Post-procedural infectious complications and communication of results

**Fig. 3:**
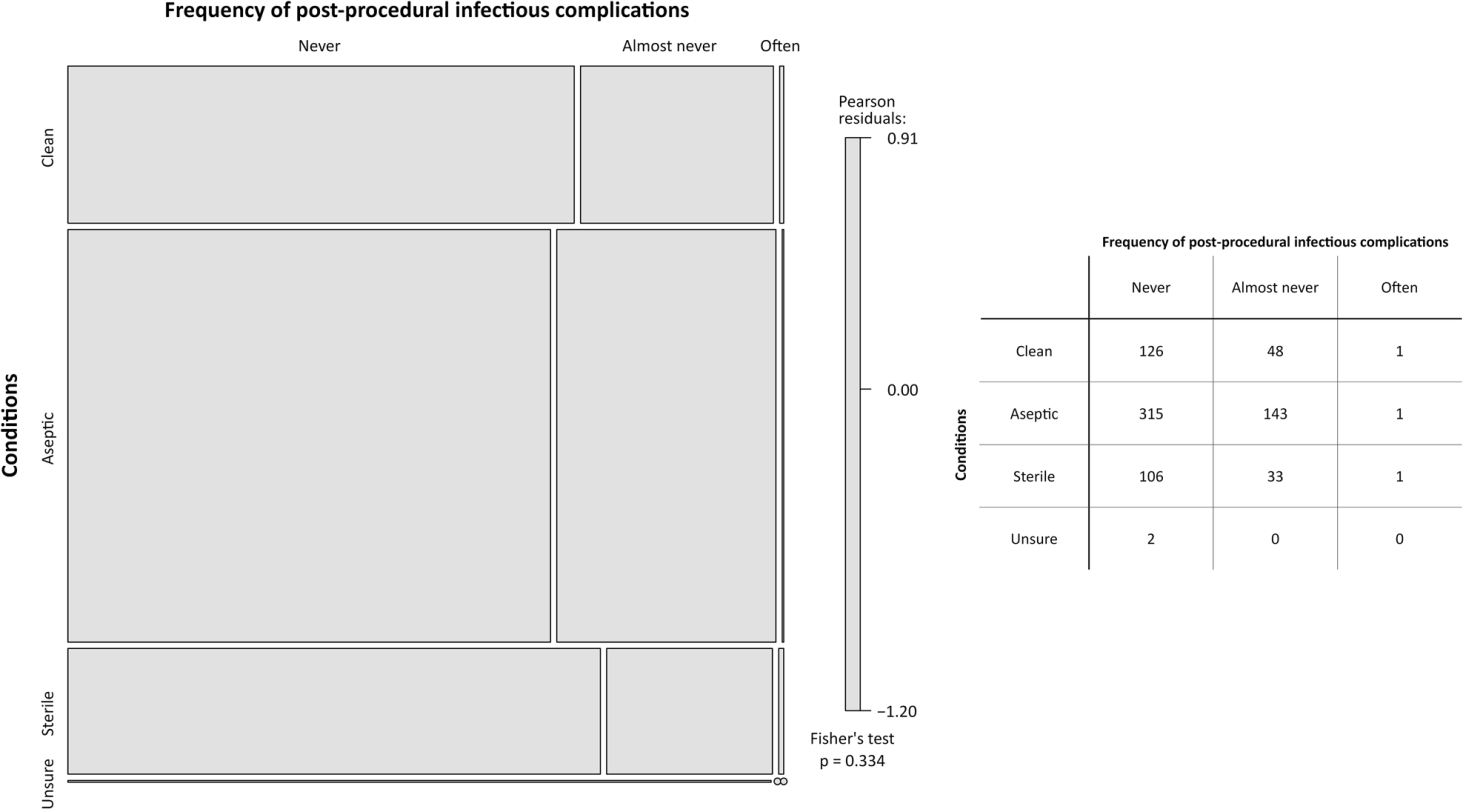
Mosaic plot and contingency table for the association between procedural conditions and the occurrence of infectious complications after ultrasound-guided core-needle breast biopsy

Among the 776 respondents who answered the question about the occurrence of infectious complications after ultrasound-guided core-needle biopsy, 549 (70.7%) declared that they had never seen any, and another 224/776 (28.9%) reported that they “almost never” saw any infectious complications. Only 3/776 respondents (0.4%) “often see” post-procedural infectious complications. Among these 776 respondents, 774 were able to categorise their procedures as clean, aseptic, or sterile (Fig. 3), without any significant association between the grade of sterility and the occurrence of post-procedural infectious complications (Fisher’s exact test *p* = 0.334).

**Fig. 4:**
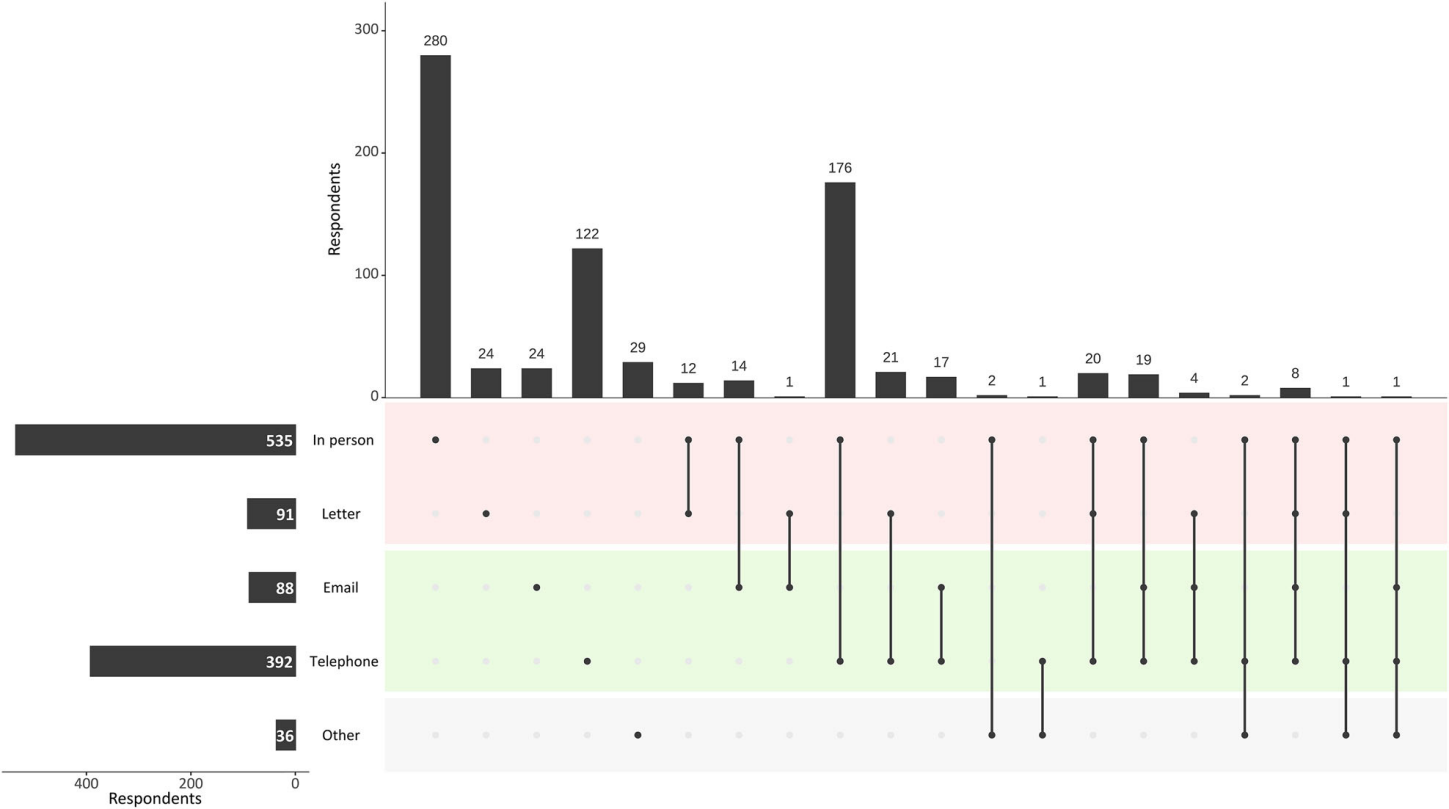
Upset plot of the respondents’ preferences for the communication of benign results after ultrasound-guided core-needle breast biopsy. Categories shaded in red indicate resource-intensive modalities

Finally, for the communication of benign biopsy results (Fig. 4), resource-intensive modalities such as in-person communication and communication by letter were used by 68.0% (535/787) and 11.6% (91/787) of respondents, respectively.

## Discussion

Over the last few years, radiology as a discipline and as a professional community has begun to devise and implement a framework to recognise, evaluate, and address its impact on the environment [8, 9, 23]. Arguably, ultrasound-guided core-needle breast biopsy represents one of the most common interventional procedures across the world, and constitutes one of the most resource-intensive acts performed in the preoperative phase of breast cancer care [11, 17]. Surprisingly, only a loose procedural framework exists for core-needle breast biopsy, which is known to be performed with widely varying protocols, especially concerning the choice of pre-procedural and peri-procedural hygiene practices and the use (and the amount) of disposable materials. This represents a paradox in a field where highly detailed quality standardisations and evaluations have been developed and enforced for diagnostic techniques such as mammography [24, 25] and indeed for the entire breast cancer care pathway [26, 27]. Moreover, this lack of procedural standardisation contributes to waste generation, which in turn results in increased expenses for waste management and for the procurement of disposable material [28].

This scenario must be accurately and consistently taken into account when interpreting and contextualising the results of this survey. As in other surveys promoted or endorsed by EUSOBI [25, 29–32], the 787 respondents who declared to routinely perform ultrasound-guided core-needle breast biopsy (out of a total 823 respondents to the survey) came from all continents and represented a broad spectrum of practice settings, from private outpatient clinics to public hospitals and academic tertiary care centres. This adds several institutional and national layers of heterogeneity to the known and expected intra-individual protocol variations. In particular, as this survey aimed to map several facets of the core-needle biopsy workflow that entail environment-related consequences—such as the use of disposable materials—resource constraints (or excessive abundance) due to country-specific or institution-specific economic conditions may have played a part in defining how breast imaging professionals effectively perform this procedure. Overall, most respondents used no more than one unit (or prepackaged bundles) of most disposable materials for each procedure: while we could not ascertain whether this indeed derives from individual consciousness about the economic costs of these materials, it indirectly translates into a resource-savvy approach.

However, a more complicated and less favourable scenario emerged when considering some aspects where personal preferences of the operators play a greater part in defining resource use and some other aspects where an active role of the operator is needed to achieve sustainable practices. For instance, while no significant association between the level of procedural sterility and the occurrence of post-procedural infectious complications (which were self-reported as practically non-existent by 70.7% of respondents) was found, up to 77.1% of respondents declared to prepare a self-reported aseptic (58.4%) or even sterile (18.7%) working environment. This is a crucial point for two reasons: first, because percutaneous core-needle biopsy is classifiable as a low-risk procedure even in basic cleanliness conditions [13, 16], therefore positioning aseptic or sterile environments as overly prudent approaches that are not supported by robust evidence; second, and most importantly, because subsequent declarations of the type and quantity of disposable materials used during the procedure point to the fact that properly defined sterility (and most likely also properly defined aseptic conditions) was most likely not attained even if it was self-reported, resulting in sizable and repeated waste of materials and also effectively nullifying the efforts towards attaining higher levels of procedural hygiene, however superfluous they might be. Likewise, several steps of the core-needle biopsy procedure where the operator must deliberately take action to ensure sustainable practices showed less favourable trends, as for instance more than a half (51.3%) of respondents declared not to use a recycle bin for the disposal of non-sharp tools, a little more than a half (53.9%) declared to avoid using prepackaged biopsy kits, and only about a fifth of the respondents (163/787, 20.7%) declared to avoid resource-intensive options for communicating negative biopsy results, such as letters or in-person appointments, thus helping to reduce unnecessary hospital visits and the associated carbon emissions from patient travel.

Aside from the geographic heterogeneity of the respondents, which might reflect differences in training and in economic conditions that directly influence the availability of materials, three main limitations of this work must be acknowledged. First, although comprising almost 800 single replies from respondents routinely performing breast biopsy, our sample undoubtedly still offers a partial representation of current practice and is likely mostly composed by EUSOBI members, who might have a relatively higher degree of acquaintance with the discourse revolving around the environmental impact of radiology, which has been widely promoted in scientific journals and international congresses over the last few years [3, 4, 8, 9, 23]. Second, this survey focused only on ultrasound-guided core-needle biopsy: while this procedure remains by far the most widely performed type of breast biopsy, the emerging role of other minimally-invasive breast interventions, such as vacuum-assisted breast biopsy and vacuum-assisted excision, deserves to be considered in future studies, as the evidence-based higher required level of hygiene for these procedures implies a correspondingly higher use of disposable materials. Third, no analyses could be conducted on how institutional factors beyond the control of each respondent directly influence their decision to use a specific amount of a disposable material during the core-needle biopsy procedure: these factors include—but are not limited to—institutional procurement contracts, standardised packaging and stockage of supplies, and local regulations about the use of pharmaceutical products or devices.

In conclusion, this survey provides a first mapping of current practices of ultrasound-guided core-needle breast biopsy that carry an environmental impact. This survey highlights wide variability in these procedural aspects, reveals that some economically driven sustainable behaviours are already in place, and shows substantial opportunities to reduce material use and waste. The absence of significant associations between sterility levels and post-procedural infectious complications suggests that resource-intensive habits—that result in avoidable waste of materials without any proven benefit for the patients—might be safely streamlined with the help of high-quality evidence and of standardised guidance.

## Supplementary information

ELECTRONIC SUPPLEMENTARY MATERIAL

## Data Availability

All data supporting the findings of this study are available within the paper.

## Abbreviation

EUSOBI: European Society of Breast Imaging

## References

[1] Or Z, Seppänen A-V (2024) The role of the health sector in tackling climate change: a narrative review. Health Policy 143:105053. 10.1016/j.healthpol.2024.105053 38537397 10.1016/j.healthpol.2024.105053

[2] Landrigan PJ, Raps H, Cropper M et al (2023) The Minderoo–Monaco Commission on plastics and human health. Ann Glob Heal 89:23. 10.5334/aogh.4056 10.5334/aogh.4056 PMC10038118 36969097

[3] Singh H, Eckelman M, Berwick DM, Sherman JD (2022) Mandatory reporting of emissions to achieve net-zero health care. N Engl J Med 387:2469–2476. 10.1056/NEJMsb2210022 36516087 10.1056/NEJMsb2210022

[4] Blom IM, Rasheed FN, Singh H et al (2024) Evaluating progress and accountability for achieving COP26 Health Programme international ambitions for sustainable, low-carbon, resilient health-care systems. Lancet Planet Heal 8:e778–e789. 10.1016/S2542-5196(24)00206-7 10.1016/S2542-5196(24)00206-7 39393379

[5] Padget M, Devadason A, Blom I et al (2024) Measuring environmentally sustainable health care: a scoping review. Lancet Planet Heal 8:e675–e683. 10.1016/S2542-5196(24)00162-1 10.1016/S2542-5196(24)00162-1 39243783

[6] Bhutta M, Rizan C (2024) The green surgery report: A guide to reducing the environmental impact of surgical care, but will it be implemented? Ann R Coll Surg Engl 106:475–477. 10.1308/rcsann.2024.0005 38683381 10.1308/rcsann.2024.0005 PMC11214859

[7] Kouwenberg LHJA, Cohen ES, Hehenkamp WJK et al (2024) The carbon footprint of hospital services and care pathways: a state-of-the-science review. Environ Health Perspect. 10.1289/EHP14754. 10.1289/EHP14754 PMC11675664 39729358

[8] Rockall AG, Allen B, Brown MJ et al (2025) Sustainability in radiology: position paper and call to action from ACR, AOSR, ASR, CAR, CIR, ESR, ESRNM, ISR, IS3R, RANZCR, and RSNA. Eur Radiol. 10.1007/s00330-025-11413-7 10.1007/s00330-025-11413-7 PMC12350511 40009087

[9] McKee H, Brown MJ, Kim HHR et al (2024) Planetary health and radiology: why we should care and what we can do. Radiology. 10.1148/radiol.240219. 10.1148/radiol.240219 38652030

[10] Braithwaite J, Smith CL, Leask E et al (2024) Strategies and tactics to reduce the impact of healthcare on climate change: systematic review. BMJ 387:e081284. 10.1136/bmj-2024-081284 39379104 10.1136/bmj-2024-081284 PMC11459334

[11] Bick U, Trimboli RM, Athanasiou A et al (2020) Image-guided breast biopsy and localisation: recommendations for information to women and referring physicians by the European Society of Breast Imaging. Insights Imaging 11:12. 10.1186/s13244-019-0803-x 32025985 10.1186/s13244-019-0803-x PMC7002629

[12] Huppe AI, Brem RF (2020) Minimally invasive breast procedures: practical tips and tricks. AJR Am J Roentgenol 214:306–315. 10.2214/AJR.19.22082 31825258 10.2214/AJR.19.22082

[13] Veltri A, Bargellini I, Giorgi L, Almeida PAMS, Akhan O (2017) CIRSE guidelines on percutaneous needle biopsy (PNB). Cardiovasc Intervent Radiol 40:1501–1513. 10.1007/s00270-017-1658-5 28523447 10.1007/s00270-017-1658-5

[14] Patel IJ, Rahim S, Davidson JC et al (2019) Society of Interventional Radiology Consensus Guidelines for the periprocedural management of thrombotic and bleeding risk in patients undergoing percutaneous image-guided interventions—part II: recommendations. J Vasc Interv Radiol 30:1168–1184.e1. 10.1016/j.jvir.2019.04.017 31229333 10.1016/j.jvir.2019.04.017

[15] Loving VA, Johnston BS, Reddy DH et al (2023) Antithrombotic therapy and hematoma risk during image-guided core-needle breast biopsy. Radiology 306:79–86. 10.1148/radiol.220548 35997610 10.1148/radiol.220548

[16] Cervini P, Hesley GK, Thompson RL, Sampathkumar P, Knudsen JM (2010) Incidence of infectious complications after an ultrasound-guided intervention. AJR Am J Roentgenol 195:846–850. 10.2214/AJR.09.3168 20858808 10.2214/AJR.09.3168

[17] Sanderink WBG, Camps-Herrero J, Athanasiou A et al (2025) Image-guided biopsy of breast lesions—when to use what biopsy technique. Insights Imaging 16:208. 10.1186/s13244-025-02084-5 40996584 10.1186/s13244-025-02084-5 PMC12463797

[18] Shum PL, Kok HK, Maingard J et al (2022) Sustainability in interventional radiology: Are we doing enough to save the environment? CVIR Endovasc 5:60. 10.1186/s42155-022-00336-9 36441364 10.1186/s42155-022-00336-9 PMC9703417

[19] de Reeder A, Hendriks P, Plug – van der Plas H et al (2023) Sustainability within interventional radiology: opportunities and hurdles. CVIR Endovasc 6:16. 10.1186/s42155-023-00362-1 10.1186/s42155-023-00362-1 PMC10027964 36939973

[20] Anneveldt KJ, Nijholt IM, Schutte JM et al (2024) Waste analysis and energy use estimation during MR-HIFU treatment: first steps towards calculating total environmental impact. Insights Imaging 15:83. 10.1186/s13244-024-01655-2 38517607 10.1186/s13244-024-01655-2 PMC10959896

[21] Anudjo MNK, Vitale C, Elshami W et al (2023) Considerations for environmental sustainability in clinical radiology and radiotherapy practice: a systematic literature review and recommendations for a greener practice. Radiography 29:1077–1092. 10.1016/j.radi.2023.09.006 37757675 10.1016/j.radi.2023.09.006

[22] James BD, Ward CP, Hahn ME, Thorpe SJ, Reddy CM (2024) Minimizing the environmental impacts of plastic pollution through ecodesign of products with low environmental persistence. ACS Sustain Chem Eng 12:1185–1194. 10.1021/acssuschemeng.3c05534 38273987 10.1021/acssuschemeng.3c05534 PMC10806995

[23] Hanneman K, Redenius I, Dewey M et al (2025) Framework for environmentally sustainable radiology: call for collaborative action and a health-centered focus. Radiology 315:e250070. 10.1148/radiol.250070 40261175 10.1148/radiol.250070

[24] Feigin K (2023) Quality assurance in mammography: an overview. Eur J Radiol 165:110935. 10.1016/j.ejrad.2023.110935 37354771 10.1016/j.ejrad.2023.110935 PMC10528604

[25] Michalopoulou E, Clauser P, Gilbert FJ et al (2023) A survey by the European Society of Breast Imaging on radiologists’ preferences regarding quality assurance measures of image interpretation in screening and diagnostic mammography. Eur Radiol 33:8103–8111. 10.1007/s00330-023-09973-7 37481690 10.1007/s00330-023-09973-7 PMC10598074

[26] Maes-Carballo M, Gómez-Fandiño Y, Reinoso-Hermida A et al (2021) Quality indicators for breast cancer care: a systematic review. Breast 59:221–231. 10.1016/j.breast.2021.06.013 34298301 10.1016/j.breast.2021.06.013 PMC8322135

[27] Rubio IT, Marotti L, Biganzoli L et al (2024) EUSOMA quality indicators for non-metastatic breast cancer: an update. Eur J Cancer 198:113500. 10.1016/j.ejca.2023.113500 38199146 10.1016/j.ejca.2023.113500

[28] White-Dzuro CE, Doyle PW, Larson MC, Frederick-Dyer KC (2025) Garbage out: a radiologist’s guide to hospital waste streams. J Comput Assist Tomogr 49:180–190. 10.1097/RCT.0000000000001681 39631737 10.1097/RCT.0000000000001681

[29] Clauser P, Mann R, Athanasiou A et al (2018) A survey by the European Society of Breast Imaging on the utilisation of breast MRI in clinical practice. Eur Radiol 28:1909–1918. 10.1007/s00330-017-5121-4 29168005 10.1007/s00330-017-5121-4 PMC5882636

[30] Trimboli RM, Capra D, Codari M, Cozzi A, Di Leo G, Sardanelli F (2021) Breast arterial calcifications as a biomarker of cardiovascular risk: radiologists’ awareness, reporting, and action. A survey among the EUSOBI members. Eur Radiol 31:958–966. 10.1007/s00330-020-07136-6 32851451 10.1007/s00330-020-07136-6 PMC7813731

[31] Lo Gullo R, Sevilimedu V, Baltzer P et al (2022) A survey by the European Society of Breast Imaging on the implementation of breast diffusion-weighted imaging in clinical practice. Eur Radiol. 10.1007/s00330-022-08833-0 10.1007/s00330-022-08833-0 PMC9064723 35507050

[32] Schiaffino S, Cozzi A, Clauser P et al (2024) Current use and future perspectives of contrast-enhanced mammography (CEM): a survey by the European Society of Breast Imaging (EUSOBI). Eur Radiol 34:5439–5450. 10.1007/s00330-023-10574-7 38227202 10.1007/s00330-023-10574-7

