# Supplementary material for: Environmental sustainability of ultrasound-guided core-needle breast biopsy: a survey on current practices by the European Society of Breast Imaging (EUSOBI)
Source: Insights Imaging. 2026 Feb 3;17:33. doi: 10.1186/s13244-026-02215-6 (PMC12868546; doi:10.1186/s13244-026-02215-6)
Supplement: Supplementary file 1 — ELECTRONIC SUPPLEMENTARY MATERIAL [file 13244_2026_2215_MOESM1_ESM.pdf]

Environmental sustainability of ultrasound-guided core-needle breast biopsy: a survey on current practices by the European Society of Breast Imaging (EUSOBI)

ELECTRONIC SUPPLEMENTARY MATERIAL

Survey questionnaire

1. Where do you work (country)?

……………….

2. Do you perform ultrasound guided breast biopsy in your daily practice?

- Yes
- No

If the respondent answered "No", the survey ended.
Respondents who answered "Yes" proceeded to the following questions.

3. Where do you perform US guided biopsy? *(Multiple choice)*

- University Hospital / Research Centre
- Public Hospital
- Private Hospital
- Private outpatient setting (self-funded/insurance)

4. How would you define how you perform US guided breast biopsies?

Sterile: Performed in a completely sterile environment to eliminate all microorganisms.
Aseptic: Use aseptic techniques (including sterile gloves) to minimize contamination.
Clean: Basic cleanliness is mantained, but not at the level of sterility or asepsis.

- Sterile procedure
- Clean
- Aseptic
- I don't know

5. Do you perform US guided biopsy with a collaborator/assistant?

- Never
- Almost never
- Often
- Always

6. Who saves images/video during the procedure? *(Multiple choice)*

- Myself with foot-switch
- Myself with keyboard
- My collaborator/assistant
- I don't save images

7. Typically, how many pairs of sterile gloves do you use during the biopsy procedure (including any assistants present)?

- 0
- 1
- 2
- 3
- >3

8. Typically, how many pairs of single use non-sterile gloves do you use during the biopsy procedure (including any assistants present)?

- 0
- 1
- 2
- 3
- >3

9. How many keyboard covers do you usually use for a procedure?

- 0
- 1
- 2
- 3
- >3

10. What do you use to cover the ultrasound probe?

- Sterile probe cover
- Non-sterile probe cover
- I do not use anything to cover the probe
- Sterile glove
- Non-sterile glove

11. How many probe covers/gloves (sterile or not) do you usually use for a procedure?

- 0
- 1
- 2
- 3
- >3

12. How many sterile drapes do you use to set up the operative field?

- 0
- 1
- 2
- 3
- 4
- 5
- >5

13. How many sterile gowns do you use during the procedure (also considering any collaborators)?

- 0
- 1
- 2
- 3
- >3

14. How many hair caps do you use during the procedure (also considering any collaborators)?

- 0
- 1
- 2
- 3
- >3

15. How many packets of sterile gel do you usually use during the procedure?

- 0
- 1
- 2
- 3
- >3
- One for more than one procedure

16. How many packs of sterile gauze do you use during the procedure (average)?

- 0
- 1-3
- 3-5
- 5-8
- 8-10
- >10

17. How many packs of non-sterile gauze do you use during the procedure (average)?

- 0
- 1-3
- 3-5
- 5-8
- 8-10
- >10

18. How many vials of local anesthetic do you usually use? *(Multiple choice)*

	0	1	2	3 or more
2 ml vial				
5 ml vial				
10 ml vial				

19. How many scalpels do you use during the procedure?

- 0
- 1
- 2
- >2

20. How many plastic containers do you use during the procedure? Plastic containers are defined as those used, for example, as containers for disinfectant/garments/cutters. *NOT THE SPECIMEN CONTAINERS.*

- 0
- 1
- 2
- 3
- >3
- One for more than one procedure

21. Do you use a biopsy kit to perform the procedures (a pre-assembled kit with multiple items inside)?

- Yes
- No

22. Do you use a recycling bin for disposal of some material during US guided biopsy?

- Yes
- No

23. How many infectious complications post breast biopsy have you seen in your experience?

- I have never seen any infectious complications post biopsy
- I almost never saw any infectious complications post biopsy
- I have often seen infectious complications post biopsy
- I don't know

24. How *BENIGN* biopsy results are communicated to the patient? *(Multiple choice)*

- Telephone
- In-person
- By e-mail
- By letter
- I don't know

25. How *MALIGNANT* biopsy results are communicated to the patient? *(Multiple choice)*

- Telephone
- In-person
- By e-mail
- By letter
- I don't know
